# Exercise for the heart: signaling pathways

**DOI:** 10.18632/oncotarget.4770

**Published:** 2015-07-23

**Authors:** Lichan Tao, Yihua Bei, Haifeng Zhang, Junjie Xiao, Xinli Li

**Affiliations:** ^1^ Department of Cardiology, The First Affiliated Hospital of Nanjing Medical University, Nanjing 210029, China; ^2^ Regeneration and Ageing Lab and Experimental Center of Life Sciences, School of Life Science, Shanghai University, Shanghai 200444, China; ^3^ Shanghai Key Laboratory of Bio-Energy Crops, School of Life Science, Shanghai University, Shanghai 200444, China

**Keywords:** exercise, cardiovascular disease, cardiac growth

## Abstract

Physical exercise, a potent functional intervention in protecting against cardiovascular diseases, is a hot topic in recent years. Exercise has been shown to reduce cardiac risk factors, protect against myocardial damage, and increase cardiac function. This improves quality of life and decreases mortality and morbidity in a variety of cardiovascular diseases, including myocardial infarction, cardiac ischemia/reperfusion injury, diabetic cardiomyopathy, cardiac aging, and pulmonary hypertension. The cellular adaptation to exercise can be associated with both endogenous and exogenous factors: 1) exercise induces cardiac growth via hypertrophy and renewal of cardiomyocytes, and 2) exercise induces endothelial progenitor cells to proliferate, migrate and differentiate into mature endothelial cells, giving rise to endothelial regeneration and angiogenesis. The cellular adaptations associated with exercise are due to the activation of several signaling pathways, in particular, the growth factor neuregulin1 (NRG1)-ErbB4-C/EBPβ and insulin-like growth factor (IGF)-1-PI3k-Akt signaling pathways. Of interest, microRNAs (miRNAs, miRs) such as miR-222 also play a major role in the beneficial effects of exercise. Thus, exploring the mechanisms mediating exercise-induced benefits will be instrumental for devising new effective therapies against cardiovascular diseases.

## INTRODUCTION

Cardiovascular disease (CVD) is the leading cause of death worldwide and exerts a considerable emotional and economic burden [1, 2]. Thus, there is an important unmet need for new cardioprotective treatments. In recent years, CVD treatments have made remarkable advances in bridging both medical and surgical approaches. Nevertheless, effective cardiovascular preventive treatments remain limited.

Various studies have demonstrated that lifestyle changes, including cessation of smoking, a healthy diet, and a regular exercise regimen can help prevent or treat CVD [3]. Among these, exercise has been reported to effectively reduce cardiovascular morbidity and mortality [4]. Recently, a study showed that compared to no physical exercise at all, any level of leisure-time physical exercise is associated with decreased rate of sudden death [5]. Those who do less exercise than recommended in the “2008 physical activity guidelines for Americans” still have a 20% lower rate of sudden death. Those people who do exercise and achieve the minimum level recommended have a 31% lower risk than those who do not exercise at all [5]. In addition, exercise has also been reported to improve functional capacity, endothelial function and collateralization in patients with diverse forms of CVD [6]. However, although it appears evident that exercise may confer a cardioprotective phenotype, the strength of the effects and the underlying mechanisms remain elusive.

The main aim of this review is to explore the beneficial effects of exercise in protecting against heart damage, with a special emphasis on the mechanisms underlying these protective effects.

## WHAT IS PROPER EXERCISE?

The relationship between physical exercise and CVD has increasingly been in the spotlight in recent years. Generally, participants engaging in exercise enjoy more desirable health outcomes, including better health-related quality of life and functional capacity [7]. However, rigorous exercise is not suited for everyone, and in these individuals, a less stringent form of exercise may help protect against CVD. So, what is proper exercise? In clinical studies, ‘proper exercise’ is defined as several months of mild-to-moderate physical activity or intensity-controlled-exercise training consisting of walking, jogging, swimming, skiing, or cycling 3 to 4 times a week [8]. The control group in these studies is advised to maintain prior exercise habits. The success of the exercise regimen is assessed by the increase in oxygen uptake (VO2) in the training group as compared to the control group [9]. The intensity of exercise is expressed as percent of VO2 max or percent of maximal heart rate (HR). Moderate intensity exercise is that performed at a relative intensity of 40% to 60% of VO2 max, while vigorous-intensity exercise is that performed over 60% of VO2 max [10]. For moderate-intensity exercise, a person's target heart rate should be 50 to 70% of his or her maximum HR. For vigorous intensity exercise, a person's target heart rate may be up to 70 to 85% of his or her maximum HR [11]. This estimate of maximum HR is based on the person's age. It can be obtained by subtracting the person's age from 220 [11]. Thus, the formula of the HR of moderate-intensity exercise is : HR = (50% to 70%)*(220-age).

For basic research using rodent models, there are two types of physical exercise utilized: wheel running and swimming. According to previous studies, both acute (i.e., 1–5 days) and chronic (i.e., weeks to months) exercise conferred benefits for CVD, especially myocardial ischemic injury [8]. However, for different models of myocardial injury, the manner, strength and length of exercise could vary for investigating the beneficial effects of exercise and underlying pathophysiological and molecular mechanisms.

## CELLULAR ADAPTATIONS IN THE CONTEXT OF EXERCISE (FIGURE 1)

**Figure 1:**
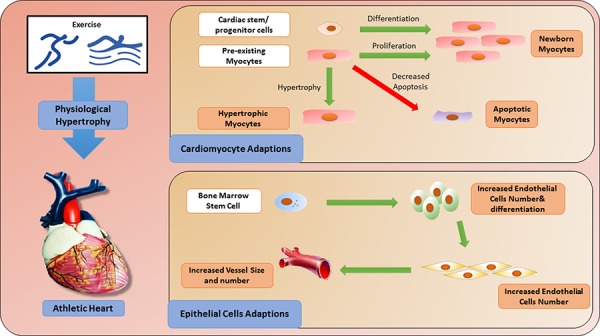
Cellular adaptations induced by exercise

### Exercise-induced cardiac growth: hypertrophy and renewal of cardiomyocytes

Physical exercise-induced cardiac growth comprises a range of changes in heart tissue including cardiac hypertrophy and renewal. Among these, the main morphological and structural adaptation in the myocardium is cardiac hypertrophy, which results in an increase in wall thickness and a growth in the size of cardiomyocytes [12]. Cardiac physiological hypertrophy in response to exercise training differs from pathological hypertrophy in its stimuli, structure and molecular profile [13]. Physiological hypertrophy occurs mainly as a result of regular physical activity or exercise training; in contrast, cardiac pathological hypertrophy occurs in response to a series of stimuli, such as myocardial infarction, valve disease and dilated cardiomyopathy. Physiological hypertrophy is characterized by normal organization of cardiac structure and normal or even elevated cardiac function, and it has a protective effect against CVD. On the other hand, pathological hypertrophy is associated with cardiac dysfuction, myocardial structural disorder, cardiac fibrosis and upregulation of fetal genes, including atrial natriuretic peptide (ANP), B-type natriuretic peptide (BNP) and skeletal α-actin and β-myosin heavy chain (MHC) [14].

Over several decades, the concept that the heart is a post-mitotic organ without any regenerative capacity had taken hold [15]. However, more recent evidence has shown that young hearts have robust growth and regenerative capacity supported by cardiomyocyte division and cardiac stem and progenitor cell activation. In fact, nearly half of the cardiomyocytes are replaced during a normal human lifespan [16]. Human cardiomyocytes retain some proliferative capacity through adulthood, which can be enhanced through physical exercise. For example, it has been reported that endurance swim training in mice induced cardiomyocyte hypertrophy and proliferation through decreased expression of the transcription factor C/EBPβ and increased expression of ED-rich carboxy-terminal domain 4 (CITED4) [17]. This exercise-induced cardiac renewal protects against pathological cardiac remodeling [17, 18].

In addition to cardiomyocyte division, the newly formed exercise-induced cardiomyocytes might also arise from cardiac stem cells (CSCs) resident in the adult heart [19–22]. CSCs, derived from embryonic stem cells or somatic stem cells [23], are self-renewing, clonogenic and multipotent. They can give rise to cardiomyocytes, smooth muscle cells and endothelial cells [24, 25]. It has been described that physical exercise could activate CSCs, thus giving rise to mature cardiomyocytes and improving the function and regeneration of the cardiovascular system [26]. Interestingly, studies have also demonstrated that the growth of smooth muscle cells and endothelial cells may be regulated by differentiated CSCs in a manner associated with exercise-induced cardiac repair [27].

Apoptosis is a specific form of programmed cell death that plays an important role in development, growth and disease [28]. It has been shown that exercise-induced cardiac growth does not result in cardiac apoptosis [29, 30], and will not induce a reactivation of cardiac fetal genes. Importantly, exercise-induced adaptive changes via cardiac hypertrophy and renewal have been demonstrated to be protective in pathological conditions such as ischemic injury. The protective effect is characterized by reduced cardiac apoptosis and decreased reactivation of cardiac fetal genes [31, 32].

### Exercise-induced effects on endothelial progenitor cells

Endothelial progenitor cells (EPCs), a type of circulating progenitor cell derived from bone marrow stem cells (BMSCs) [33, 34], can be activated in response to physical exercise [35]. The importance of EPCs in the context of CVD has been well documented, with the number and function of EPCs being correlated with cardiovascular risk factors [36] and the number of circulating EPCs serving as a predictor of cardiovascular events and death [37]. In addition, during cardiac injury, especially AMI, BMSCs can trans-differentiate into vascular endothelial cells and cardiomyocytes to implant into damaged mycardium and improve cardiac function [38]. Exercise can induce EPCs to proliferate, migrate and differentiate into mature endothelial cells [35] which promote endothelial regeneration and angiogenesis. Thus, the increased concentration of EPCs in response to physical exercise may serve as a physical repair or compensatory mechanism in the setting of cardiac injury.

Overall, cellular adaptations induced by exercise can be attributed to both endogenous and exogenous factors. Physical training switches on the endogenous system and promotes cardiomyocyte self-renewal and cellular turnover. This is of potential importance as it serves as the basis for regeneration of an injured heart [39]. Nevertheless, it is still unknown to what extent the CSCs and/or the proliferation of resident cardiomyocytes contribute to the generation of new cardiomyocytes of the adult mammalian heart [40]. Meanwhile, exercise also promotes an exogenous system, favoring not only the repair of subclinical lesions but also mobilizing exogenous cells against harmful conditions [41–43]. Among exogenous cell types, endothelial cells are the most important ones. For example, exercise reduces the incidence of atherosclerotic heart disease and may promote the formation of vascular branches [44]. Interestingly, since cardiomyocytes are regulated by physical exercise, they can also affect other cell types, such as endothelial cells and smooth muscle cells [45]. Cardiomyocytes and other cell types may be complementary in the context of exercise to improve cardiac function and repair.

## EXERCISE PROTECTS AGAINST CARDIOVASCULAR DISEASES (TABLE 1)

**Table 1:** Exercise protects against cardiovascular diseases

Types of disease	Effects of exercise	References
Myocardial Infarction	1. Decrease inflammation 2. Reduce cardiac apoptosis 3. Decrease cardiac fibrosis	45–51
Ischemia/Reperfusion Injury	1. Up-regulate anti-oxidative defense 2. Promote angiogenesis 3. Decrease cardiac apoptosis	52–57
Diabetic Cardiomyopathy	1. Enhance mitochondrial biogenesis 2. Improve vascular endothelial function	58–60
Cardiac aging	1. Decrease cardiac apoptosis 2. Increase antioxidant defence 3. Decrease cardiac fibrosis	61–65
Atherosclerosis	1. Promote vascular branch formation 2. Regulate mitochondrial biogenesis	44
Pulmonary hypertension	1. Increase aerobic capacity 2. Raise muscle strength 3. Enhance exercise tolerance 4. Improve quality of life	66–69

### Exercise protects against myocardial infarction

Acute myocardial infarction (AMI) is a prevailing cause of death worldwide. Cardiac remodeling after AMI refers to changes in the size, shape, structure and composition of the heart [46]. Such injuries result in loss of cardiomyocytes by programmed cell death and are accompanied by compensatory induction of hypertrophic growth and fibrosis. Recent large-scale studies have provided support for the notion that exercise protects against AMI remodeling both in patients and animal models [47–49].

Inflammation plays an important role in AMI-associated cardiac remodeling, and exercise is an effective means to modulate inflammation-associated cytokine activities. Exercise has been shown to reduce pro-inflammatory cytokines levels including C-reactive protein (CRP), IL-1, IL-6 and INF-γ, and increase anti-inflammatory cytokines levels such as IL-10. This blunts inflammation and improves the coronary risk profiles [48, 50]. Besides its effect on inflammation, exercise is also effective in reducing cardiac apoptosis by regulating the mitochondrial-mediated apoptotic pathway, which is assessed via lower terminal deoxynucleotidyl transferase-mediated dUTP nick end labeling (TUNEL)-positive staining, caspase-3 cleavage and a decreased ratio of Bax (pro-apoptotic)/Bcl-2(anti-apoptotic) protein levels [51]. Additionally, exercise can also reduce myocardial infarct size and fibrosis by down-regulating the fibrosis-related factor TGF-β [49]. Other potential mechanisms underlying the protective effect of exercise against AMI include increased regeneration of cardiomyocytes, metabolic regulation and decreased risk of re-infarction.

### Exercise protects against cardiac ischemia/reperfusion injury

Cardiac ischemia/reperfusion (I/R) Injury is defined as myocardial damage when oxygenated blood returns to the heart after a period of ischemia or lack of oxygen [52]. The absence of oxygen or nutrients from blood creates a condition in which the restoration of circulation results in apoptosis and oxidative damage through the induction of oxidative stress [52]. Previous studies have demonstrated that exercise training can protect against I/R injury in clinical patients and SD rats [53].

Oxidative stress is an important component participating in I/R injury, which is characterized by accumulation of reactive species and damage in the heart and other organs such as the lung and brain. Studies have demonstrated that regular exercise leads to up-regulation of anti-oxidative defense mechanisms, which help minimize oxidative stress following I/R [54]. For example, during five weeks of five weekly exercise sessions, an increased antioxidant capacity has been observed in the heart. The prevention of excessive nitric oxide synthesis limited its binding to O2 and consequent formation of peroxynitrite [55]. Exercise also induces EPCs to promote angiogenesis. The growth of new blood vessels is an important natural process required for healing wounds and for restoring blood flow to tissues after I/R injury [56]. Finally, during the process of I/R, there is significant cardiac dysfunction and myocardial apoptosis [57]. Exercise results in decreased apoptosis and improved cardiac function by increasing the expression of AKT, AKT phosphorylation and glycogen synthase kinase (GSK)-3β phosphorylation. Treatment with a PI3K kinase inhibitor abolished the beneficial effects of exercise, providing mechanistic insights into the effects of exercise on cardiac apoptosis and function [53].

### Exercise protects against diabetic cardiomyopathy

Diabetic cardiomyopathy (DCM) is a disorder of the heart muscle that occurs in the setting of type I or type II diabetes. DCM can compromise the ability of the heart to effectively pump and circulate blood throughout the body and can lead to fluid accumulation in the lungs or legs [58]. DCM is a major cause of morbidity and mortality among diabetics, encompassing structural, morphological, functional, and metabolic abnormalities in the heart. Recently, it has been shown that exercise can protect against DCM, especially in type II diabetes [59].

Mitochondrial dysfunction is one of the major mechanisms underlying DCM. DCM associated-downregulation of PGC-1α and its downstream components leads to decreased mitochondrial biogenesis [59]. Increasing evidence has shown that exercise can increase the number and size of mitochondria by upregulating PGC-1α and nuclear respiratory factor 1 (NRF-1) signaling pathways. Improved mitochondrial biogenesis can also increase the efficient oxidation of carbohydrates, thus improving glucose tolerance and insulin sensitivity [60]. Notably, exercise has been shown to improve cardiac mitochondrial biogenesis in DCM due to activation of PGC-1α, providing compelling evidence of exercise-associated protection for cardiac metabolism [59].

### Exercise protects against cardiac aging

Progressive aging induces several structural and functional disorders in the cardiovascular system characterized by increased myocyte size, reduced number of cardiomyocytes and increased interstitial collagen fibers [61]. A growing literature suggests that exercise has a positive effect on cardiovascular performance in both elderly patients and animal models of aging [62, 63].

Aging-related cardiomyocytes loss is mediated by apoptosis and necrosis. The mitochondrial-mediated apoptotic pathway in particular is the best characterized and believed to play a critical role in regulating apoptosis due to aging [62]. As discussed earlier, exercise can decrease the level of apoptosis in cardiac and skeletal muscles of young adult rats by changing the expression of apoptosis regulatory factors such as Bcl-2 and apoptotic protease activating factor-1 (apaf-1) [64]. Physical exercise training may also increase the antioxidant defense and decrease oxidative stress in the elderly. Furthermore, exercise protects against aging-associated cardiac fibrosis by reducing fiber production via downregulated TGF-β1, which is a potential contributor to fibrosis, in addition to increasing collagen degradation via increased MMP (MMP-1, MMP-2, MMP-3, and MMP-4) and decreased TIMP expression [65].

### Exercise protects against pulmonary hypertension

Pulmonary hypertrophy (PH) is a disease with increased blood pressure in the pulmonary arteries, pulmonary veins or capillaries. PH can cause a number of symptoms including shortness of breath, dizziness, fainting and leg swelling [66]. More recently, a body of literature has shown the safety and efficacy of exercise training in PH.

In clinical studies, exercise training has been identified to confer numerous clinical benefits in patients with PH, including increasing aerobic capacity, rasing muscle strength, enhancing exercise tolerance and ultimately improving quality of life [67]. Exercise also plays a key role in a subtype of PH, pulmonary artery hypertension (PAH). In rats, PAH or right ventricular failure can be induced by a single injection of monocrotaline (MCT). Voluntary exercise by running has been demonstrated to retain exercise capacity and delay the onset of heart failure, thereby improving the survival rate in rats with PAH [68, 69]. However, even though the positive phenotype of exercise on PH or PAH is obvious, the underlying mechanism needs to be studied in further detail.

### Molecular mechanisms underlying exercise-induced beneficial effects in the heart

**Figure 2:**
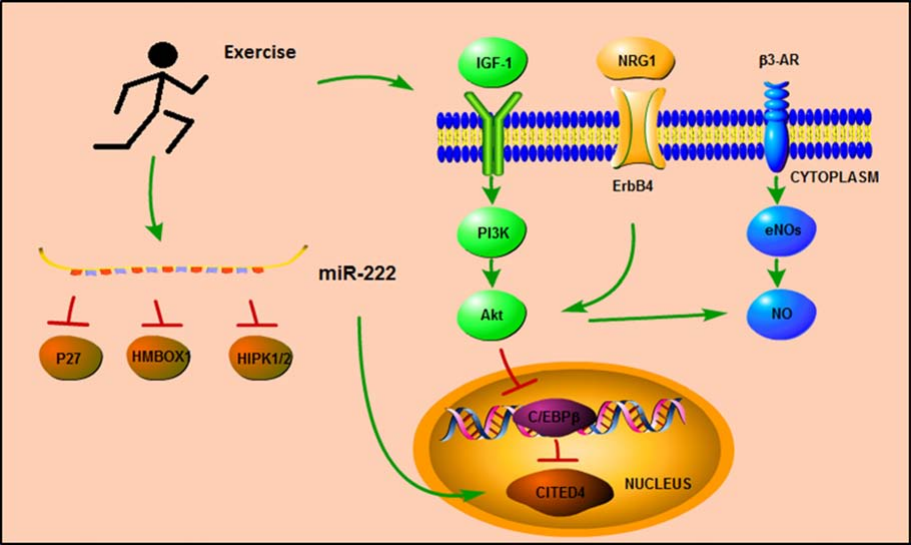
Signaling pathways mediates in exercise training

Exercise-induced cardiac protection has been appreciated for many decades, and several molecular mechanisms have been proposed to mediate exercise-associated cardioprotective phenotypes (Figure 2).

### Signaling pathways underlying protective effects of exercise

Growth factor neuregulin1 (NRG1)-ErbB4-C/EBPβ is one of the main pathways implicated in mediating the changes in cardiomyocytes induced by exercise [70, 71]. The effect of exercise on cardiomyocyte hypertrophy and proliferation involves reduction in the expression of the transcription factor C/EBPβ, and a linked increase in the expression of CITED4 [17]. The reduction of C/EBPβ expression level results in upregulation of hypertrophy-related genes, such as Gata4, Tbx5, Nkx2.5, α-MHC, TnI, and TnT [17]. Of note, Gata4 was recently reported to regulate cell proliferation during cardiac regeneration [72]. Moreover, C/EBPβ is also an important functional target of NRG1-related signaling pathways. Injection of NRG1 in adult mice induces cardiomyocyte proliferation and promotes myocardial regeneration, leading to improved function following myocardial infarction [73].

The insulin-like growth factor (IGF)-1-PI3k-Akt signaling pathway also plays an important role in the protective effects conferred by regular physical exercise [74, 75]. Exercise increases cardiac expression of IGF-1, and treatment with IGF-1 up-regulates myocardial telomerase activity and increases expression of phosphorylated Akt protein kinase, thereby regulating cardiac hypertrophy, viability and homeostasis.

AMP-activated protein kinase (AMPK) is an enzyme participating in cellular energy homeostasis. Recently, exercise was identified to be one of the factors upregulating AMPK, and the activation of AMPK is also responsible for most of the benefical effects resulting from physical exercise [76]. One study demonstrated that swimming training decreased isoproterenol (ISO)-induced cardiac fibrosis by inhibiting the ROS-NADPH oxidase pathway mediated by AMPK activation [77]. Another study also reported that activated AMPK induced by endurance exercise conferred positive effects against type 2 diabetes by increasing fatty acid and glucose metabolism, mitochondrial content and insulin sensitivity. Interestingly, the benefical effects of exercise may also be related to PGC-1α, another key factor which is also increased in exercise training [78].

Endothelial nitric oxide synthase (eNOS) and nitric oxide (NO) seem to be closely related to the positive effects of exercise on endothelial cells [79, 80]. Interestingly, exercise alters the expression and phosphorylation status of eNOS in a tissue-specific manner. In the heart, exercise increases the expression of eNOS-PSer1177 and decreases the expression of eNOS-PThr495 without changing the expression of total eNOS, whereas in the skeletal muscle, exercise increases the expression of total eNOS and decreases the expression of eNOS-PThr495 [81]. Recent studies also reported that the activation of vascular eNOS during exercise could be caused by several signaling pathways involving Akt (protein kinase B), protein kinase A (PKA) and/or AMPK, and inhibition of Akt signaling decreases the expression of eNOS-PSer1177 in mice subjected to endurance exercise in comparison to vehicle-treated exercised mice, but without changing the expression of phosphorylated CREB (PKA signaling) or AMPK [82]. Previous studies demonstrated that β3-adrenergic receptors (β3-AR) played a major role in regulating the phosphorylation of eNOS at serine 1177 as well as in maintaining the basal expression of myocardial eNOS during exercise [81, 83]. The relationship between β3-AR, eNOS, and Akt during exercise may yield promising insights into therapeutic targets against myocardial injury.

### MicroRNAs responsible for cardiac protection effects of exercise (Table 2)

**Table 2:** microRNAs in response to exercise training

MicroRNAs	Targets	Function	References
miR-1, miR-133a, miR-133b (down)	RhoA, Cdc42, NELFA	1. Increase cell differentiation & growth	88–90
miR-214 (down)	SERCA2a	1. Increase left ventricular myocyte mass 2. Increase cell contraction	91
miR-29a, miR-29c(up)	COL1A1, COL3A	1. Increase ventricular compliance 2. Decrease cardiac collagen	92, 93
miR-222 (up)	P27, HMBOX1, HIPK1/2	Increase cardiac growth	94, 95
miR-126 (up)	MAPK PI3K/Akt/eNOS	Increase cardiac angiogenesis	97

MicroRNAs (miRNAs, miRs) are highly-conserved, non-protein coding RNAs which can inhibit target gene expressions and functions by binding to the 3′ untranslated region (3′ UTR) of messenger RNA (mRNA) and thereby repressing their translation and/or promoting their degradation [84]. miRNAs regulate cardiac development, hypertrophy, and angiogenesis [85–87]. miR-1, -133a, and -133b are the well-known miRNAs explored in heart [88, 89]; these three families are down-regulated in three cardiac models of hypertrophy including transverse aortic constriction (TAC), Akt transgenic mice and endurance exercise. In addition, during 12 weeks of physical exercise, miR-1, -133a, and -133b were shown to decrease cell differentiation and growth by inhibiting Ras homolog gene family-A (RhoA), cell division control protein 42 (Cdc42) and wolf-Hirschhorn syndrome candidate (NELFA) [90]. These data indicate that miR-1, -133a, and -133b have a close relationship with cardiac hypertrophy both in physiological and pathological contexts, with further investigation required to document the function of these three miRNA families in cardiac hypertrophy. Another miRNA, miR-214, is also decreased upon resistance training, leading to increased left ventricular myocyte width and volume and faster cell contraction in rats during eight weeks of training. SERCA2a, a target gene of miR-214, was increased in the exercise group [91].

Recent reports document that physical training induces up-regulation of the expression of miR-29a and -29c in the heart, which are associated with a significant decrease in left ventricu1ar collagen (COLIAI and COLIIIAI) gene and protein levels [92]. The up-regulation of the miR-29 family is also accompanied by improvement of ventricular compliance and cardiac function [93].

More recently, miR-222 has been reported to be an important mediator of exercise-induced cardiac growth by inhibiting Cyclin-dependent kinase inhibitor 1B (p27), Homeobox containing 1 (HMBOX1) and Homeodomain-interacting protein kinase 1/2 (HIPK1/2). In ischemic injury models, miR-222 protects against adverse cardiac remodeling and cardiac dysfunction [94]. Interestingly, miR-222 has also been linked to CITED4; the up-regulation of miR-222 in swimming mice is associated with increased expression of CITED4, indicating cross-interaction between miR-222 and the C/EBPβ/CITED4 signaling pathway [95].

The expression of miR-126 has also been shown to be increased in aerobic training in rats during ten weeks of swimming, leading to cardiac angiogenesis via targeting MAPK and PI3K/Akt/eNOS [96]. Similarly, miR-150 has been reported to be upregulated while miR-26b and -143 downregulated in the heart upon exercise [97]. However, the specific relationship and mechanisms underlying changes in miRNA levels upon physical exercise still need to be studied in further detail.

In addition to local miRNAs, plasma-based “circulating” miRNAs (c-miRNAs) are significantly changed in the setting of regular exercise in humans, including miR-106a, -30b, -146, -338 and -21 [96, 98, 99]. c-miRNAs may become potent biomarkers of physiological exercise and provide novel insight into the physical exercise process.

## CONCLUSION

In this review, we summarize the protective effects of exercise against cardiac injury and address the underlying cellular and molecular mechanisms. Exercise increases myocardial oxygen supply and extraction, reduces myocardial fibrosis and cardiomyocyte apoptosis, promotes angiogenesis, and also improves cardiac function and cardiomyocyte survival in various CVD. For the cellular mechanisms, we provide an overview of both exercise-induced cardiac hypertrophy and renewal, and exercise-induced mobilization of EPCs to proliferate, migrate and differentiate into mature endothelial cells capable of regeneration and angiogenesis as primary contributors. For the molecular mechanisms, we summarize C/EBPβ-Cited4 and IGF-1-PI3k-Akt as the main signaling pathways mediating the changes in cardiomyocytes induced by exercise. Exercise also has established relationships with miRNAs, with recent reports documenting that exercise induced upregulation of miRNAs-29a and -29c correlates with a significant decrease in left ventricular collagen gene and protein concentration [92]. In addition, the expression of miR-222 was increased upon exercise to regulate myocyte growth via targeting of p27, HMBOX1, and HIPK1/2. Also, downregulation of miRNA-1, -133a, -133b and -214 were observed in the exercised heart [92]. Thus, the protective effects of exercise against myocardial damage are clearly pleiotropic in their mechanisms.

Although evidence of the protective effects of physical exercise against cardiac injury is strong, certain caveats need to be noted. First, the optimal level of exercise for preventing CVD is unclear. In some studies, the reduction in risk from increased levels of exercise activity appeared to be linear up to a certain level, above which there was no further benefit; in others, the effect was restricted to the highest categories of total energy expenditure [100]. The response of the heart is not dependent on the type of exercise applied, but rather on the duration and intensity at which the exercise is performed [101]. Moderate versus vigorous exercise may have completely different effects following myocardial damage. There is an urgent need to determine to what extent exercise can ameliorate disease processes and reduce morbidity and mortality. Likewise, the origin of cardiomyocyte regeneration induced by exercise is still elusive.

In summary, exercise is a potent functional intervention to protect against CVD. Future studies are needed to explore the specific strength and intensity of exercise required to protect against cardiac injuries, to investigate which cell types specifically contribute to exercise-induced cardiac adaptations, and to define the complex interplay between these cell types.
